# Comparative Genomics Reveals Novel Species and Insights into the Biotechnological Potential, Virulence, and Resistance of *Alcaligenes*

**DOI:** 10.3390/genes14091783

**Published:** 2023-09-10

**Authors:** Francisnei Pedrosa-Silva, Thiago M. Venancio

**Affiliations:** Laboratório de Química e Função de Proteínas e Peptídeos, Centro de Biociências e Biotecnologia, Universidade Estadual do Norte Fluminense Darcy Ribeiro, Campos dos Goytacazes 28013-602, Brazil; francisneipedrosa@gmail.com

**Keywords:** bioinformatic, bacteria, biotechnology

## Abstract

*Alcaligenes* is a cosmopolitan bacterial genus that exhibits diverse properties which are beneficial to plants. However, the genomic versatility of *Alcaligenes* has also been associated with the ability to cause opportunistic infections in humans, raising concerns about the safety of these microorganisms in biotechnological applications. Here, we report an in-depth comparative analysis of *Alcaligenes* species using all publicly available genomes to investigate genes associated with species, biotechnological potential, virulence, and resistance to multiple antibiotics. Phylogenomic analysis revealed that *Alcaligenes* consists of at least seven species, including three novel species. Pan-GWAS analysis uncovered 389 species-associated genes, including cold shock proteins (e.g., *csp*A) and aquaporins (e.g., *aqp*Z) found exclusively in the water-isolated species, *Alcaligenes aquatilis*. Functional annotation of plant-growth-promoting traits revealed enrichment of genes for auxin biosynthesis, siderophores, and organic acids. Genes involved in xenobiotic degradation and toxic metal tolerance were also identified. Virulome and resistome profiles provide insights into selective pressures exerted in clinical settings. Taken together, the results presented here provide the grounds for more detailed clinical and ecological studies of the genus *Alcaligenes*.

## 1. Introduction

The genus *Alcaligenes* belongs to the family *Alcaligenaceae* and consists of motile, Gram-negative, and rod-shaped or coccus-shaped bacteria. The bacteria of this genus are widely found in various environments, such as soil, water, plants, and hospital settings. Some *Alcaligenes* species exhibit plant-beneficial properties and have demonstrated a capacity to promote plant growth through production of siderophores [1,2], to promote the solubilization of phosphate, and to antagonize phytopathogenic microorganisms [1]. In addition, members of the *Alcaligenes* genus have demonstrated the capacity to remove trace metals such as Cadmium [3,4] and degrade toxic pollutants (e.g., phenol) [5], polyaromatic hydrocarbons (PHAs) [6], and pesticides [7,8,9], demonstrating potential agricultural and industrial benefits.

In contrast to its closely related sister genus *Bordetella*, *Alcaligenes* species are typically non-pathogenic. However, some species have been associated with opportunistic infections [10,11]. The type species of the genus, *Alcaligenes faecalis*, has been associated with nosocomial infections and was detected in clinical samples such as blood, respiratory secretions, and urine [12,13]. In some cases, *A. faecalis* infections are difficult to treat due to high resistance to multiple antibiotics [13,14,15].

In recent decades, several isolates of *Alcaligenes* spp. have been extensively investigated. A comparative genome analysis of *A. aquatilis* QD168 and 25 other strains of *Alcaligenes* spp. identified gene features relating to abiotic stress and aromatic compound degradation [16]. Recently, a comparison of *Alcaligenes* sp. Mc250 and 13 *Alcaligenes* spp. genomes revealed genes of biotechnological interest, including denitrification, benzene degradation, and metabolism of metals such as zinc, cadmium, and arsenic [17]. Although these characteristics have been investigated in some isolates, the phylogenetic relationships within *Alcaligenes* are still not fully explored, especially in the context of biofertilization, bioremediation, and resistance to multiple antibiotics.

Here, we report a comprehensive comparative genomic analysis of *Alcaligenes*, which allowed us to identify new species and uncover important features of the phylogenetic relationships, biotechnological potential, virulence, and resistance profiles of the different species of the genus.

## 2. Materials and Methods

### 2.1. Dataset and Genome Curation

We downloaded 2492 *Alcaligenaceae* family genomes from the NCBI Genbank database in May 2023. The genome distance estimation analysis of *Alcaligenaceae* genomes was performed using Mash v.2.2.1 [18] and a family-wide distance network was generated using the R package igraph v.0.10.5. *A. faecalis* DSM 30030 type strain was used as reference to find *Alcaligenes* genomes with maximum Mash distances of 0.15 (~85% average nucleotide identity (ANI)) [19]. Genome quality was evaluated with CheckM v.1.0.13 [20], using a minimum of 90% completeness and a maximum of 10% contamination. Genomes with more than 500 contigs were removed, and contigs smaller than 500 bp were removed from the remaining genomes. In order to remove near-identical redundant genomes, we used *in house* scripts to cluster genomes with pairwise Mash distances smaller than 0.005 (~99.95% ANI) and keep the one with the greatest N50 as the cluster representative. Genome-wide nucleotide identity values were estimated using all-against-all ANI based on MUMmer alignment (ANIm) with pyANI v.0.27 [21]. 

### 2.2. Genomic Features and Phylogeny

All *Alcaligenes* genomes were re-annotated with Prokka v.1.12 [22] to avoid bias in the identification of protein families. Plasmids and genomic islands were predicted with Plasforest v.1.4.0 [23] and Islandviewer4 (www.pathogenomics.sfu.ca/islandviewer/ accessed on 2 August 2023) [24], respectively. Orthologous genes were identified with Orthofinder v.2.5.5 [25]. All predicted single-copy orthologous genes present in all isolates were used for maximum-likelihood phylogenetic analysis with IQ-tree v.2.0.5 [26], using the best-fit model, JTT + F + I + G4, inferred with ModelFinder. Bootstrap support values were estimated with the ultrafast bootstrap method with 1000 replicates [27]. The resulting phylogenetic tree was visualized and rendered with iTOL v4 [28]. 

### 2.3. Pangenome Analysis

The *Alcaligenes* pangenome was computed with Roary v.3.6, using an 80% identity threshold to determine gene clusters [29]. The pangenome-wide association study (pan-GWAS) analysis was performed with Scoary v.1.6.16 [30], using the Roary output to establish which genes were typical of *Alcaligenes* groups containing at least five genomes, while correcting for population structure using the phylogenetic tree. False-discovery rate was estimated by Benjamini–Hochberg-adjusted *p*-value provided in Scoary. We only reported the results with specificity > 90% and Benjamini–Hochberg-corrected *p*-value  ≤ 0.05. The heatmaps of trait-associated genes were rendered using the R package tidyverse v.1.3.1 [31]. 

### 2.4. Plant-Growth-Promoting Traits Analysis

Genes associated with plant-growth-promoting traits (PGPT) and of biotechnological interest were predicted using Usearch v.11.0.667 [32] to search the Plant-associated bacterium database, PLaBAse (www.plabase.cs.uni-tuebingen.de accessed on 21 May 2023) [33], with minimum 50% and 80% identity and coverage thresholds, respectively. Comparison of PGPT number was plotted as a z-scaled heatmap using the R package tidyverse.

### 2.5. Resistome and Virulome Analysis

Antimicrobial resistance and virulence genes were predicted using Usearch v.11.0.667 to search the *Alcaligenes* genomes against the Comprehensive Antibiotic Resistance Database (CARD) (www.card.mcmaster.ca/ accessed on 14 January 2023) and the Virulence Factors of Pathogenic Bacteria Database (VFDB) (http://www.mgc.ac.cn/ accessed on 15 January 2023) databases, respectively. Minimum identity and coverage thresholds of 50% and 80% were used in these searches, respectively. The presence/absence profiles of virulence and resistance-associated genes were rendered using the R package tidyverse v.1.3.1.

## 3. Results and Discussion

### 3.1. Data Selection and Genus Classification

**Figure 1:**
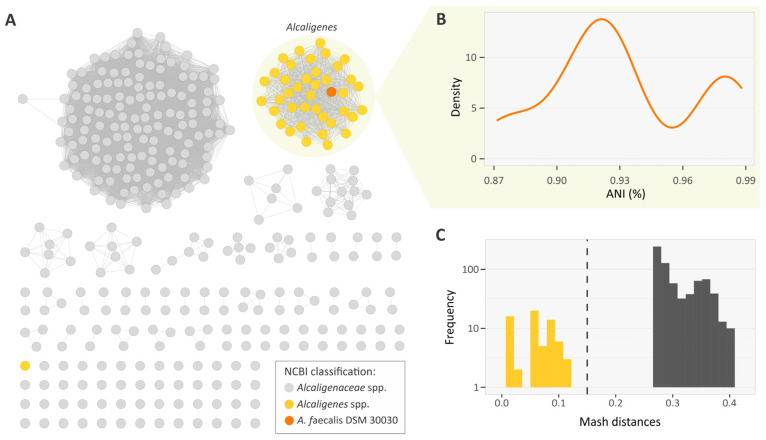
Genomic diversity of *Alcaligenaceae* family. (**A**) Mash-distance-based network of *Alcaligenaceae*, built using 2492 publicly available genomes. The *Alcaligenes* community is shaded in yellow. (**B**) Density plot of pairwise ANI of *A. faecalis* DSM 30030 with *Alcaligenes* community. (**C**) Mash distance between 2492 *Alcaligenaceae* genomes and the type strain *A. faecalis* DSM 30030. The maximum Mash distance threshold (0.15) used to select genomes is represented by dotted line. The Mash-distance values of *Alcaligenes* community is shaded in yellow.

To accurately compare *Alcaligenes* with minimal genome misclassification, we retrieved 2492 Alcaligenaceae genomes from GenBank (April 2023). We then filtered out low-quality, fragmented, and redundant genomes (see methods). Using Mash v.2.2.2 [18], we computed pairwise distances between genomes to generate a Mash distance network with a minimum threshold of 0.20 (~80% ANI). Networks inferred with genomic distance or identity are highly structured and feature communities associated with taxonomic groups [19]. We used the type strain *A. faecalis* DSM 30030 (GCF_002443155.1) as an anchor isolate to evaluate Mash distances in the Alcaligenaceae family network (Figure 1A). This identified a community of 64 genomes corresponding to the *Alcaligenes* genus according to the NCBI classification. The ANI analysis showed densities above 87% (Figure 1B), and the sorted distribution of Mash values for *A. faecalis* DSM 30030 showed an abrupt break around 0.13 (Figure 1C), suggesting that 0.15 (~85% ANI) is an effective threshold for delineating the *Alcaligenes* genus. The type strain *A. endophyticus* DSM 100498 (GCA_026344035) was removed as a misclassified *Alcaligenes* genome. The final dataset of 64 high-quality *Alcaligenes* genomes was used in the downstream analyses described below.

### 3.2. Phylogenetic Analysis of Alcaligenes

**Figure 2:**
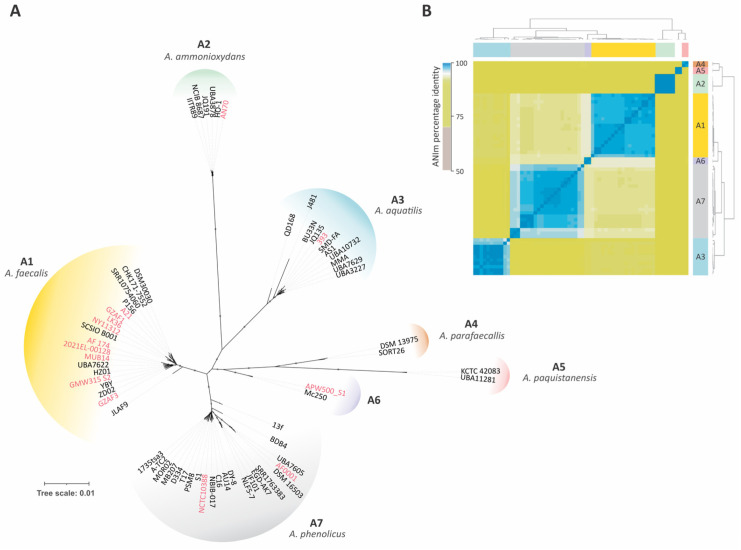
Phylogeny analysis and genomic diversity of *Alcaligenes* genus. (**A**) Phylogenetic tree of *Alcaligenes* genomes showing seven groups. A total of 1172 single-copy orthologous genes were used to build a maximum likelihood phylogenetic tree using IQ-tree (see methods for details). Clinical genomes are highlighted in red. (**B**) Pairwise ANI values between the 65 *Alcaligenes* genomes.

In order to uncover the phylogenetic relationships among the *Alcaligenes* genomes, we performed a maximum-likelihood phylogenetic reconstruction using the proteins encoded by 1272 single-copy orthologous genes inferred with Orthofinder v.2.5.5. Our analysis revealed that *Alcaligenes* comprises at least seven highly supported phylogenetic groups (A1 to A7) (Figure 2A). This result is supported by ANIm analysis, which showed genomic identity above 95% between the genomes of each group and allowed us to hypothesize that each group comprises an *Alcaligenes* species (Figure 2B).

The *Alcaligenes* group A1 corresponds to *A. faecalis* and contains 19 strains, including the type strain *A. faecalis* DSM 30030 (Table 1). The phylogenetic group A2 corresponds to a recently characterized species, *A. ammonioxydans* [34], and comprises six strains, including four misclassified strains (*A. faecalis* AN70, *A. faecalis* subsp. *phenolicus* IITR89, *A. faecalis* subsp. *faecalis* NCIB 8687, and *A. faecalis* UBA7838). *Alcaligenes* group A3 corresponds to *A. aquatilis* and contains 11 strains, including 7 misclassified strains (i.e., *A. faecalis* J481, *A. faecalis* JQ135, *A. faecalis* UBA10732, *A. faecalis* UBA3227, *A. faecalis* UBA7629, *Alcaligenes* sp. SMD-FA, and *Alcaligenes* sp. MMA). The phylogenetic group A5 corresponds to *A. pakistanensis* and includes two strains, the type strain *A. pakistanensis* KCTC 42083 and the misclassified strain *A. faecalis* UBA 11281. The *Alcaligene*s group A6 represents a potentially novel species with two isolates (*A. faecalis* MB250 and *A. faecalis* APW500_S1). Finally, the groups A4 andA7 comprise 2 and 22 misclassified strains, respectively. Based on the presence of the recently reclassified *A. faecalis* subsp. *parafaecalis* DSM 13975 and *A. faecalis* subsp. *phenolicus* DSM 16503 [35], and also supported by ANI analysis, we propose to designate these groups as *Alcaligenes parafaecalis* and *Alcaligenes phenolicus*, respectively, and reclassify all genomes accordingly. Further, clinical isolates were identified in all groups, except for A4 and A5. Furthermore, *A. faecalis* has the largest frequency of clinical isolates, corresponding to 47% (9) of *Alcaligenes* group A1.

### 3.3. Pangenome and Pan-GWAS Analyses of Alcaligenes

Aiming to better understand genomic traits and dynamics at the genus level, we computed the *Alcaligenes* pangenome. The pangenome is defined as the total number of non-redundant genes present in a given set of genomes [36]. In our analysis, 9444 gene clusters were identified. The core genome comprises 2686 genes present in at least 90% of the genomes. These genes correspond to 28.44% of the pangenome and are typically associated with intrinsic physiological traits.

**Figure 3:**
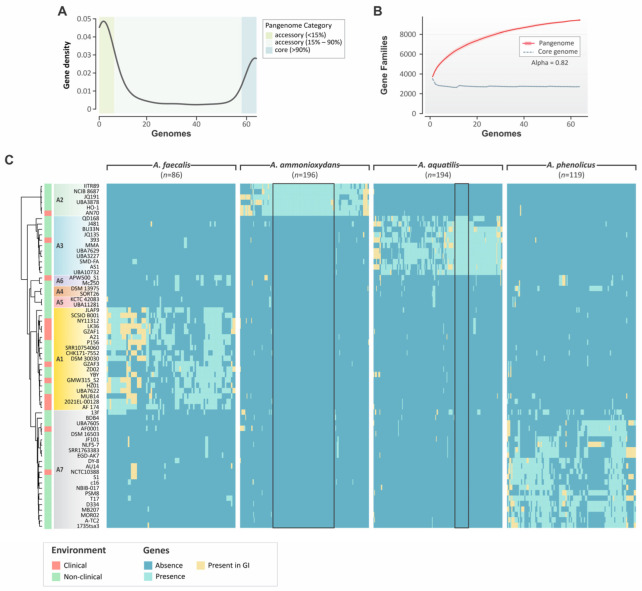
Pangenome and pan-GWAS of *Alcaligenes*. (**A**) Gene frequency of the *Alcaligenes* pangenome. (**B**) Number of gene families in the *Alcaligenes* pangenome. The cumulative curve (in red) and α value of Heaps’ law (0.82) supports an open pangenome. (**C**) Distribution of species-associated genes in *Alcaligenes*. The heatmaps represent the presence or absence of the genes identified by the pan-GWAS pipeline using *Alcaligenes* groups (species) as traits. The black square highlights the most strongly associated genes found for each trait (i.e., 100% specificity and sensitivity).

The accessory genome is composed of 1733 high-frequency genes (present in 15% to 90% of the genomes) and 5025 low-frequency genes (present in up to 15% of the genomes), corresponding to 18.35% and 53.20% of the pangenome (Figure 3A), respectively. The abundance of low-frequency genes further reflects the genome plasticity of *Alcaligenes* and probably plays a role in niche adaptation between species. The Heaps’ law estimate supports an open pangenome (α = 0.48), indicating a high level of genetic diversity and allowing us to predict that many more additional gene clusters will be detected as new genomes are sequenced (Figure 3B). The open pangenome of *Alcaligenes* is also in line with previous reports [17,37]. Although the *Alcaligenes* pangenome analyses reported here help us understand the evolution and genomic dynamics of the genus, a clearer picture will be available only when a significant number of genomes become sequenced for each species (i.e., phylogenetic group), as divergent genome fluidity and pangenome openness estimates can be found in species from the same genus [38]. 

We conducted a pan-GWAS analysis to find accessory genes significantly associated with *Alcaligenes* groups containing at least five isolates. We found a total of 595 genes, consisting of 86, 196, 194, and 119 genes associated with the A1, A2, A3, and A7 groups, respectively (Figure 3C, Supplementary Table S1). Interestingly, 69.91% of these genes encode hypothetical proteins. Further, *A. faecalis* (A1) and *A. phenolicus* (A7) were strongly associated (100% of sensitivity and specificity) with a single gene each, both encoding hypothetical proteins. Further, the gene associated with the A1 group has a conserved UDP-glucose/GDP-mannose dehydrogenase domain (PF03721), which plays a crucial role in the biosynthesis of polysaccharides in bacteria [39]. Further, pan-GWAS analysis revealed important genes for *A. ammonioxidans* (A2) and *A. aquatilis* (A3), mainly related to stress resistance. *A. ammonioxydans* presented 105 strongly associated genes, including genes involved in amino acid transport (*sfp*, *aax*C and *asp*T), nitrosative stress resistance (*hmp*, *nor*R), and salinity stress resistance through trehalose biosynthesis (*ots*AB). Further, 25 genes were strongly associated with *A. aquatilis*, including a low-conductance mechanosensitive channel (*yna*I), an aquaporin Z (*aqp*Z), and a cold-shock protein (*csp*A). The *yna*I gene is related to cell protection against hypoosmotic stress [40], *aqp*Z encodes a channel that mediates rapid water influx or efflux in response to abrupt osmolarity changes [41], and the *csp*A gene encodes a highly conserved DNA-binding protein that is released upon abrupt temperature downshifts [42]. The differential presence of genes involved in resistance to osmotic stress and low temperatures in *A. aquatilis* appears to be associated with the aquatic lifestyles of seven isolates from this species (70%) and thus has the potential to be used as molecular markers to differentiate this species in the genus. Nevertheless, the analysis of a larger number of genomes is warranted to validate this hypothesis. 

### 3.4. Functional Annotation with Plant-Associated Bacterium Database 

**Figure 4:**
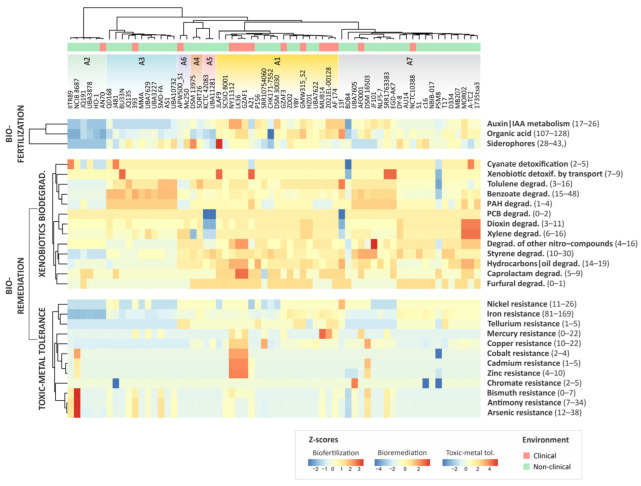
Functional plant-growth-promoting traits (PGPT) of *Alcaligenes* based on PlaBase annotations. Heatmap highlighting PGPT abundance differences in functional classes and major genetic traits of *Alcaligenes* genomes. Reddish color indicates enriched and bluish color indicates decreased number of genes based on a trait-specific z-scale. The *Alcaligenes* phylogenetic tree was placed at the top of the heatmap to allow a more comprehensive interpretation of the results.

*Alcaligenes* species have the potential to promote biofertilization, bioremediation, and heavy metal tolerance [8,9,43,44,45,46,47]. We investigated the potential of *Alcaligenes* species for these properties by integrating data from PGPTs available in the PlaBAse database. We found no differences in the PGPT counts from clinical and non-clinical genomes, suggesting that the presence of genes associated with plant growth promotion is not suitable to discriminate between beneficial and potentially pathogenic isolates. However, comparative analysis revealed gene enrichment for biofertilization traits in *A. faecalis* (A1) and *A. phenolicus* (A7), while *A. ammonioxydans* (A2) is depleted of such traits (Figure 4). The main genes related to these PGPTs are described in the following sections.

### 3.5. The Plant Growth Promotion Potential of Alcaligenes

Indole-3-acetic acid (IAA) is an auxin-class phytohormone and one of the most important plant growth regulators, known to be produced by several rhizobacteria [48]. All the genes required for the synthesis of tryptophan, the IAA precursor, were identified in all *Alcaligenes* genomes (Supplementary Figure S1). Tryptophan biosynthesis begins with anthranilate synthase (TrpEG) which catalyzes the conversion of chorismate into anthranilate. Anthranilate is converted into indole-3-glycerol phosphate in three steps by anthranilate phosphoribosyltransferase (TrpD), phosphoribosylanthranilate isomerase (TrpF), and indole-3-glycerol phosphate synthase (TrpC). Finally, tryptophan synthase (TrpAB) acts in the last step, converting this compound into tryptophan [49,50]. The bacterial IAA biosynthesis can occur by at least four tryptophan-dependent pathways, classified according to their intermediates: indole-3-pyruvic acid (IPyA), tryptamine (TRY), indole-3-acetamide (IAM), and indole-3-acetonitrile (IAN) [51]. We found that almost all *Alcaligenes* genomes (except those of the CHK171-7552 and BDB4 strains) have the indole-3-acetamide hydrolase-encoding gene (*iaa*H) that catalyzes the last step of IAM to IAA conversion (Supplementary Figures S1 and S2). Further, we identified nitrilase (*nit*A) in all genomes, suggesting that *Alcaligenes* produces IAA via the IAN pathway.

Phosphate is often a limiting factor for plant growth because it is largely insoluble in the soil, making it unavailable to plants. In this context, some plant-growth-promoting rhizobacteria (PGPR) solubilize unavailable inorganic phosphate through the secretion of organic acids [52]. We identified a set of 107 to 128 genes in *Alcaligenes* involved in the biosynthesis of 19 different organic acids (Supplementary Figure S1), including citric acid (*glt*A, *acn*AB, *prp*C), oxalic acid (*mdh*, *ace*B *dct*A), and malic acid (*fum*AB, *fum*C), reported as the most effective in phosphate solubilization by *A. faecalis* [47]. 

Iron is an essential micronutrient for plant growth, required in a number of vital processes such as respiration and photosynthesis. PGPR can enhance iron uptake by plants through the secretion of siderophores that chelate soil iron with high affinity [53]. We found 39 genes involved in the biosynthesis and transport of siderophores, including the *ent*ABCEF operon that encodes an enterobactin-like siderophore in *Escherichia coli* [54,55].

### 3.6. Biodegradation of Xenobiotic Compounds 

We found 97 genes associated with bioremediation (as per PlaBase classifications) in *Alcaligenes*, encompassing 14 PGPT pathways linked to biodegradation and metabolism of xenobiotics, including dioxin, styrene, naphthalene, polychlorinated biphenyls (PCBs), hydrocarbon, polycyclic aromatic hydrocarbons (PAH), nitro derivatives of aromatic compounds, and benzene derivatives such as benzoate, toluene, and xylene (Figure 4). The *A. pakistanensis* (A5) and *A. phenolicus* 13f genome presented a low gene count for the degradation of xylene (6) and dioxin (3) and no genes for PCB degradation. This could be attributed to the absence of two key enzymes involved in phenol degradation: 4-hydroxy-2-oxovalerate aldolase (*mph*E) and acetaldehyde dehydrogenase (*mph*F). Additionally, the cis-biphenyl dihydrodiol dehydrogenase (*bph*B) and HOPDA hydrolase (*bph*D), which are involved in the PCB degradation pathway, were also absent.

*A. phenolicus* was first described as an *A. faecalis* subspecies with the ability to degrade phenolic compounds [5]. The degradation of benzoate and intermediates can be initiated via the aerobic pathway by the action of monooxygenase or dioxygenase, leading to the formation of catechol, protocatechuate, or gentisate [56]. Genes encoding monooxygenase (*pox*ABCDEF) and dioxygenase (*ben*ABCDE), and genes related to the degradation of catechol intermediates (*cat*ABCD) and protocatechuate (*pca*C, *pca*D, *pca*I, *pca*J, *pca*K) were found in all *Alcaligenes* genomes (Supplementary Figure S3). Additionally, we identified a set of genes involved in the degradation of protocatechuate derivatives (*lig*AB, *lig*I, *lig*J) in all genomes of *A. aquatilis* and in three genomes of *A. phenolicus* (DSM16503, EGD-AK7, and UBA7605), suggesting greater versatility in the degradation of benzoate and PAHs intermediates in these species. Furthermore, *A. phenolicus* MO02 presented two genes for ethylbenzene dioxygenase (*ebd*B and *ebd*C), suggesting their capacity to conduct aerobic ethylbenzene degradation [57].

Interestingly, all PHA-related genes (*nah*, *oph*E and *pht*5) that we found in *A. phenolicus* BDB4, reported as a potential PHA-degrading bacterium [6], were present in the *Alcaligenes* core genome, thus representing an intrinsic feature of the genus. Compared with other *Alcaligenes* genomes, the BDB4 strain exhibited a reduced repertoire of copies of *cyn*X (2) and *ecs*AB (7), which are associated with cyanate bioaccumulation and xenobiotic efflux, respectively.

### 3.7. Toxic Metal Tolerance

Bacteria have also become an important component in the remediation of soil and aquatic environments contaminated with trace metals such as nickel, chromium, tellurium, arsenic, and mercury [58]. Further, the utilization of metal-tolerant PGPR has proven critical for improving agricultural production in soils contaminated with trace metals [59,60]. Genes associated with nickel (*ddp*ABCDF), chromate (*chr*B, *chr*C, *chrR*), and tellurium (*act*P) resistance were found in all *Alcaligenes* genomes. The *A. faecalis* LK36, *A. faecalis* GKAF1, and *A. phenolicus* NCIB_8687 genomes showed greater enrichment for PGPT, conferring resistance to cadmium, cobalt, and zinc due to the presence of the *czc*D, *cad*C, and *czr*A genes, involved in the regulation of and tolerance against these trace metals (Figure 4) [61,62]. Arsenic resistance genes (*ars*) involved in arsenic detoxification were identified in all *Alcaligenes* genomes (Supplementary Figure S4). 

Mercury is one of the most toxic pollutants in the environment [63]. Thirteen genomes (20%) of *A. faecalis* and *A. aquatilis* harbor mercury resistance (*mer*) genes (Supplementary Figure S4). Interestingly, most of these isolates (except *A. aquatilis* UBA7629, *A. aquatilis* SMD-FA, *A. faecalis* SRR10754060, and *A. phenolicus* SRR1763383 are from clinical settings. Through a more detailed analysis, we found that these *mer* genes are located in putative genomic islands or plasmids containing antimicrobial resistance (AMR) genes. The *mer* operon encodes a common mercury resistance mechanism that is often carried by transposons and a wide range of plasmids that are ubiquitous in Proteobacteria [64,65,66]. Previous studies have reported significant co-occurrence of *mer* operon and AMR genes in mobile elements in a range of isolates, demonstrating that mercury facilitates the selection of multidrug-resistant strains [67,68,69]. Thus, the acquisition of mercury resistance genes by *Alcaligenes* isolates is likely adaptive in clinical settings. 

### 3.8. Virulence Genes and Their Distribution across Alcaligenes Species

We systematically investigated the repertoire of virulence factors using the VFDB database. In addition, we investigated the presence of these genes in genomic islands and plasmids. The *Alcaligenes* virulome comprises 71 genes (Supplementary Table S2), of which 48 (67.6%) were found in the core virulome (present in at least 90% of the genomes). No core virulome genes were detected in GI or plasmids. The core virulome comprises genes involved in motility, adherence, biofilm, iron uptake, and immune system evasion, including the *tvi*B and *tvi*C genes, related to resistance against phagocytosis through biosynthesis of VI capsular polysaccharide [70]. We also identified genes involved in oxidative stress response, including *sod*B and *sod*CI, which encode an iron and copper/zinc superoxide dismutases (SOD), respectively. *sod* genes encode important metallo-oxidoreductases that convert superoxide radicals into hydrogen peroxide and molecular oxygen and have been shown to neutralize toxic levels of reactive oxygen species generated by a range of hosts, including plants and humans [71]. For example, SOD is crucial for the endophytic colonization of rice roots by *Glucanocetobacter diazotrophicus* [72]. In humans, the *sod*CI gene has been associated with the survival of pathogenic bacteria upon the oxidative burst caused by phagocytes during infection [73,74]. Thus, the presence of *sod*CI in *Alcaligenes* might also be involved in the virulence in human hosts.

**Figure 5:**
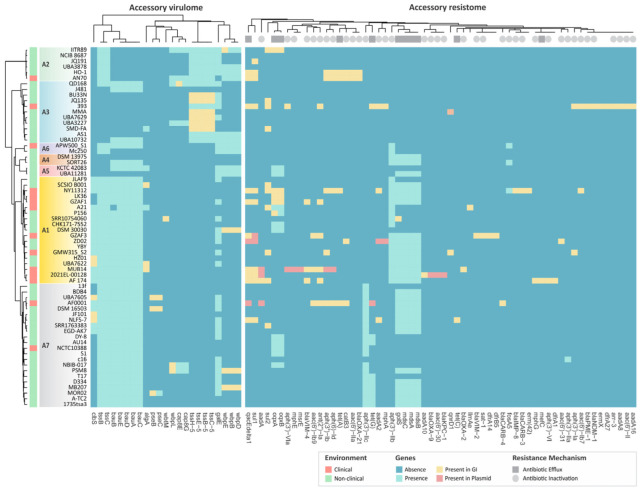
Acquired virulome and resistome of *Alcaligenes*. The respective phylogenetic groups of *Alcaligenes* are highlighted in the tree. The heatmaps represent the presence or absence of the genes identified in this work (see methods for details).

The accessory virulome (genes present in up to 90% of genomes) comprises 23 genes related to the type VI secretions system (T6SS), biofilm, exotoxin, immune modulation, and iron uptake (Supplementary Table S2). The acinetobactin gene cluster and T6SS genes were differentially distributed across *Alcaligenes* species (Figure 5). The siderophore acinetobactin acts as the major iron uptake mechanism in *Acinetobacter baumannii* [75]. Receptors that mediate the recognition and internalization of ferric-acinetobactin complexes are encoded by the *bau*ABCDE operon, which were found in 75% of the *Alcaligenes* genomes, including all the *A. faecalis* and *A. phenolicus* genomes (Figure 5). Conversely, in *A. aquatilis* and *A. ammonioxydans*, *bau*ABCDE is rare or even absent, respectively. 

T6SS is a potent weapon for interbacterial competition by delivering toxins into prokaryotic cells [76]. We found that the T6SS-HSI genes (*tss*B and *tss*C), which encode tubule-forming proteins, are absent in *Alcaligenes* group A5, *A. parafaecalis*, and *A. aquatilis* (A3, except in *A. aquatilis* QD168). Conversely, only *A. aquatilis* and *A. ammonioxydans* have the T6SS-5 genes (*tss*B-5, *tss*C-5, *tss*E-5, and *tss*H-5). Interestingly, most of the *A. aquatilis* T6SS-5 genes are present in genomic islands, indicating their acquisition through horizontal gene transfer.

### 3.9. Resistance Profiles of Clinical and Non-Clinical Genomes

The resistome analysis revealed 60 antimicrobial resistance genes in *Alcaligenes* (Supplementary Table S3). The core resistome of the genus comprises only one gene, a class A β-lactamase with an average of 54% amino acid sequence identity with BlaSCO-1, a carbenicillin-hydrolyzing β-lactamase (CARB) identified in *E. coli* and *Acinetobacter* spp. [77,78]. However, while BlaSCO-1 is generally found in plasmids [79,80], this core carbenicillinase is not found in mobile elements in *Alcaligenes*. The presence of CARB-type enzymes corroborates the observed intrinsic penicillin resistance of *Alcaligenes*, as shown for other Gram-negative bacteria [81,82]. 

The *Alcaligenes* accessory resistome has 59 genes, most of them (54) distributed at low frequency (in up to 20% of genomes) (Figure 5). The main acquired resistance mechanism in *Alcaligenes* involves antibiotic inactivation enzymes (62.3%) (Supplementary Table S3). Further, the genomes of *A. faecalis*, *A. phenolicus*, *A. parafaecalis*, and the *Alcaligenes* group A6 harbor different chromosomal aminoglycoside 3′-phosphotransferase genes (*aph*(3′)-IIb and *aph*(3′)-IIc), which confer resistance against several important aminoglycosides (e.g., kanamycin, neomycin, and seldomycin), constituting an intrinsic core resistance mechanism of these groups.

Drug efflux systems play a major role in the resistance of most Gram-negative pathogenic bacteria [83]. In contrast to *Achromobacter*, a pathogenic and phylogenetically closely related genus, *Alcaligenes* has a reduced repertoire of efflux pumps [84,85,86]. We identified nine genes related to antibiotic efflux, including *mds*ABC and *gol*S, which encode the efflux pump MdsABC and its regulator. The *mds*ABC and *gol*S were found in *Alcaligenes* groups (except in *A. aquatilis*, *A. ammonioxydans* and *Alcaligenes* A6 group), potentially conferring resistance against different antibiotics, such as β-lactams and phenicol [87,88]. Further, the operon *oqx*AB, found in *A. pakistanensis*, *A. faecalis*, and *A. phenolicus*, encodes an efflux pump that mediates resistance to multiple antibiotics such as tetracycline, nitrofuran, and fluoroquinolone [89]. We also identified the antiseptic resistance gene *qac*Edelta1, involved in efflux of quaternary ammonium compounds (e.g., disinfecting agents and antiseptics) [90]. Interestingly, *qac*Edelta1 is more prevalent in clinical genomes of *A. faecalis* and *A. aquatilis* (Figure 5), probably due to the high selection pressure imposed by antiseptics and disinfectants in clinical settings. In addition, this gene co-occurs with *sul*1, which confers resistance to sulfonamide. This combination is common in some pathogenic species such as *Klebsiella pneumoniae* e *Salmonella enterica* [91,92]. 

**Figure 6:**
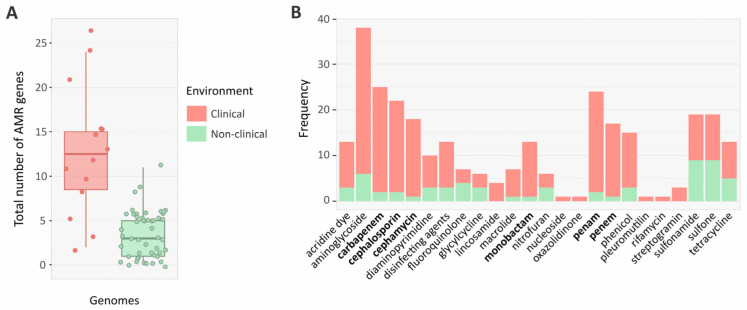
Distribution of antimicrobial resistance (AMR) genes in clinical and non-clinical isolates. (**A**) Total AMR genes identified in *Alcaligenes*. (**B**) Frequency of different classes of AMR genes in mobile genetic elements of clinical and non-clinical genomes. β-lactams are marked in bold.

Our results reveal that clinical genomes (*n* = 14) harbor around three times more AMR genes than non-clinical genomes (*n* = 50), with averages of 12 and 4 genes, respectively (Figure 6A). The clinical genome of *A. aquatilis* (strain 393) had the greatest number of acquired resistance genes (18). Interestingly, all these genes are within GIs, strongly suggesting their acquisition via HGT. Further, low-frequency resistance genes were mostly found near mobile genetic elements in the genomes of clinical isolates (Figure 6B). These genes confer resistance to a range of antibiotic classes, mainly aminoglycosides (*aac*, *aad*, *ant*, and *aph* genes), sulfonamides (*sul*1 and *sul*2), and β-lactams (*bla*CARB-3, *bla*CARB-4, *bla*IMP-8, *bla*OXA-2, *bla*OXA-9, *bla*OXA-21, *bla*VIM-2, *bla*VIM-4, *bla*KPC-1, *bla*NDM-1. *bla*PME-1, and *bla*VIM-2). Genes encoding resistance against other antibiotic classes, including rifamycin (*arr*-3), nucleoside (*sat*-1), and lincosamide (*llm*Ae), were nearly absent in *Alcaligenes*.

Acquired resistance of *Alcaligenes* has been reported sporadically, mostly associated with multidrug-resistant strains of *A. faecalis*, such as those reported by Huang [14] in bloodstream, urinary tract, skin, and soft tissue infections. In most cases, *A. faecalis* isolates were resistant to conventional antibiotic classes such as aminoglycosides, β-lactams, and macrolides [14], an observation that is supported by our results showing the presence of AMR genes conferring resistance to these antibiotics in *A. faecalis*. On the other hand, the low frequency of genes encoding resistance to rifamycin, nucleosides, and lincosamides in the *Alcaligenes* resistome suggests that these antibiotics might be good candidates to investigate as new therapeutic alternatives against pandrug-resistant *Alcaligenes* infections.

## 4. Conclusions

In this study, we report the comparative analysis of *Alcaligenes* genomes isolated from clinical and non-clinical environments. By assessing genome identity and reconstructing phylogeny, we properly assign *Alcaligenes* genomes to seven different species. Using a pan-GWAS approach, 471 genes were specifically associated with species that contained more than five isolates. *A. ammonioxydans* and *A. aquatilis* had exclusive sets of genes associated with different stress resistances and can be used as biomarkers in future studies. Functional annotation based on PGPT levels showed enrichment of traits for biofertilization and bioremediation in the *A. faecalis*, *A. phenolicus,* and *Alcaligenes* A4 group. In addition, the functional genes related to xenobiotic degradation and heavy metal tolerance are also discussed in detail. The *Alcaligenes* genomes comprise a low number of virulence and resistance genes, although most resistance genes have been associated with clinical genomes. Overall, our results provide a snapshot of the genetic diversity of the genus *Alcaligenes* and pave the way for more detailed clinical and ecological investigations.

## Figures and Tables

**Table 1:** *Alcaligenes* isolates used in this study and their respective phylogenetic group.

Accession	Strain	NCBI Classification	Reclassified as	Source	Group	Type
GCA_000967305.2	ZD02	*A. faecalis*	*-*	Nematode	A1	Non-clinical
GCA_001641975.2	P156	*A. faecalis*	*-*	Soil	A1	Non-clinical
GCA_002119995.1	GZAF3	*A. faecalis*	*-*	*Homo sapiens*	A1	Clinical
GCA_002120075.1	GZAF1	*A. faecalis*	*-*	*Homo sapiens*	A1	Clinical
GCA_002443155.1	DSM 30030	*A. faecalis*	*-*	Activated sludge	A1	Non-clinical
GCA_002484125.1	UBA7622	*A. faecalis*	*-*	Wood	A1	Non-clinical
GCA_003122065.1	YBY	*A. faecalis*	*-*	Activated sludge	A1	Non-clinical
GCA_003939865.1	AF_174	*A. faecalis*	*-*	Hospital washroom sink	A1	Clinical
GCA_008373885.1	LK36	*A. faecalis*	*-*	*Homo sapiens*	A1	Clinical
GCA_010092625.1	MUB14	*A. faecalis*	*-*	*Homo sapiens*	A1	Clinical
GCA_015905185.1	A21	*A. faecalis*	*-*	*Homo sapiens*	A1	Clinical
GCA_016446305.1	SCSIO B001	*A. faecalis*	*-*	Fungi	A1	Non-clinical
GCA_018066525.1	2021EL-00128	*A. faecalis*	*-*	*Homo sapiens*	A1	Clinical
GCA_018682645.1	HZ01	*A. faecalis* subsp. *faecalis*	*A. faecalis*	Contaminated culture of *M. chubuense*	A1	Non-clinical
GCA_019836945.1	GMW315_S2	*A. faecalis*	*-*	Hospital effluent	A1	Clinical
GCA_020741625.1	CHK171-7552	*A. faecalis*	*-*	Poultry feces	A1	Non-clinical
GCA_023921285.1	JLAF9	*A. faecalis*	*-*	Chicken manure	A1	Non-clinical
GCA_027595045.1	NY11312	*A. faecalis*	*-*	*Homo sapiens*	A1	Clinical
GCA_946479345.1	SRR10754060	*A. faecalis*	*-*	Plant	A1	Non-clinical
GCA_000275465.1	NCIB 8687	*A. faecalis* subsp. *faecalis*	*A. ammonioxydans*	-	A2	Non-clinical
GCA_001516865.1	IITR89	*A. faecalis* subsp. *phenolicus*	*A. ammonioxydans*	River water	A2	Non-clinical
GCA_002392125.1	UBA3878	*A. faecalis*	*A. ammonioxydans*	Wood	A2	Non-clinical
GCA_004319585.1	AN70	*A. faecalis*	*A. ammonioxydans*	*Homo sapiens*	A2	Clinical
GCA_019343455.1	HO-1	*A. ammonioxydans*	*-*	Wastewater	A2	Non-clinical
GCA_022436505.1	JQ191	*A. ammonioxydans*	*-*	Soil	A2	Non-clinical
GCA_002242175.1	JQ135	*A. faecalis*	*A. aquatilis*	Wastewater	A3	Non-clinical
GCA_002362965.1	UBA3227	*A. faecalis*	*A. aquatilis*	Metal/plastic	A3	Non-clinical
GCA_002484005.1	UBA7629	*A. faecalis*	*A. aquatilis*	Metal	A3	Non-clinical
GCA_003076515.1	BU33N	*A. aquatilis*	*-*	Sediment	A3	Non-clinical
GCA_003511485.1	UBA10732	*A. faecalis*	*A. aquatilis*	-	A3	Non-clinical
GCA_003671915.1	QD168	*A. aquatilis*	*-*	Marine sediment	A3	Non-clinical
GCA_003716855.1	J481	*A. faecalis*	*A. aquatilis*	Salt marsh sediment	A3	Non-clinical
GCA_003938225.2	393	*A. aquatilis*	*-*	Hospital washroom sink	A3	Clinical
GCA_020907215.1	MMA	*Alcaligenes* sp.	*A. aquatilis*	River water	A3	Non-clinical
GCA_023373785.1	AS1	*A. aquatilis*	*-*	Activated sludge	A3	Non-clinical
GCA_025960365.1	SMD-FA	*Alcaligenes* sp.	*A. aquatilis*	Sludge	A3	Non-clinical
GCA_017377875.1	SORT26	*Alcaligenes* sp.	A. *parafaecalis*	Residential yard	A4	Non-clinical
GCA_026344135.1	DSM 13975	*A. faecalis* subsp. *parafaecalis*	A. *parafaecalis*	Water	A4	Non-clinical
GCA_003521065.1	UBA11281	*A. faecalis*	*A. pakistanensis*	-	A5	Non-clinical
GCA_014652815.1	KCTC 42083	*A. pakistanensis*	*-*	Industrial wastewater	A5	Non-clinical
GCA_009497775.1	Mc250	*A. faecalis*	*Alcaligenes* sp.	Plant	A6	Non-clinical
GCA_019693795.1	APW500_S1	*A. faecalis*	*Alcaligenes* sp.	Hospital effluent	A6	Clinical
GCA_000429385.1	DSM 16503	*A. faecalis* subsp. *phenolicus*	*A. phenolicus*	Wastewater bioprocessor	A7	Non-clinical
GCA_000465875.3	EGD-AK7	*Alcaligenes* sp.	*A. phenolicus*	Soil	A7	Non-clinical
GCA_000770015.1	MOR02	*A. faecalis*	*A. phenolicus*	Nematode	A7	Non-clinical
GCA_001530325.1	NBIB-017	*A. faecalis*	*A. phenolicus*	Soil	A7	Non-clinical
GCA_002082085.1	MB207	*A. faecalis* subsp. *phenolicus*	*A. phenolicus*	Tannery effluent	A7	Non-clinical
GCA_002205415.1	BDB4	*A. faecalis*	*A. phenolicus*	Soil	A7	Non-clinical
GCA_002476455.1	UBA7605	*A. faecalis*	*A. phenolicus*	Wood	A7	Non-clinical
GCA_005311025.1	AU14	*A. faecalis*	*A. phenolicus*	Plant	A7	Non-clinical
GCA_016807785.1	c16	*A. faecalis*	*A. phenolicus*	Wastewater	A7	Non-clinical
GCA_020496585.1	13f	*Alcaligenes* sp.	*A. phenolicus*	Soil	A7	Non-clinical
GCA_022343965.1	T17	*A. faecalis*	*A. phenolicus*	Sediment	A7	Non-clinical
GCA_023101245.1	D334	*A. faecalis*	*A. phenolicus*	Mangrove	A7	Non-clinical
GCA_023702805.1	PSM8	*A. faecalis*	*A. phenolicus*	Dumpsite	A7	Non-clinical
GCA_024134565.1	1735tsa3	*Alcaligenes* sp.	*A. phenolicus*	Clean room	A7	Non-clinical
GCA_024266725.1	NLF5-7	*Alcaligenes* sp.	*A. phenolicus*	Wastewater	A7	Non-clinical
GCA_024584725.1	DY-8	*A. faecalis*	*A. phenolicus*	Soil	A7	Non-clinical
GCA_024654895.1	AF0001	*A. faecalis*	*A. phenolicus*	*Homo sapiens*	A7	Clinical
GCA_026344155.1	A-TC2	*Alcaligenes* sp.	*A. phenolicus*	Nematode	A7	Non-clinical
GCA_026799675.1	S1	*A. faecalis*	*A. phenolicus*	Soil	A7	Non-clinical
GCA_029000325.1	JF101	*A. faecalis*	*A. phenolicus*	Undersea mud	A7	Non-clinical
GCA_900445215.1	NCTC10388	*A. faecalis* subsp. *faecalis*	*A. phenolicus*	*Homo sapiens*	A7	Clinical
GCA_937863365.1	SRR1763383	*A. faecalis*	*A. phenolicus*	Wastewater	A7	Non-clinical

## Data Availability

Data and materials are available in Supplementary Information. All other relevant data are available from the corresponding author upon reasonable request.

## Supplementary Materials

The following supporting information can be downloaded at: https://www.mdpi.com/article/10.3390/genes14091783/s1, Figure S1: Distribution of biofertilization genes in *Alcaligenes*; Figure S2: Deduced pathways for IAA biosynthesis in *Alcaligenes*; Figure S3: Distribution of xenobiotic degradation genes in Alcaligenes; Figure S4: Distribution of heavy metal resistance genes in *Alcaligenes*. Table S1: Pan-GWAS analysis of *Alcaligenes* groups; Table S2: Virulome analysis of *Alcaligenes* e; Table S3: Resistome analysis of *Alcaligenes*.
