# Supplementary material for: Comparative Genomics Reveals Novel Species and Insights into the Biotechnological Potential, Virulence, and Resistance of Alcaligenes
Source: Genes (Basel). 2023 Sep 10;14(9):1783. doi: 10.3390/genes14091783 (PMC10530903; doi:10.3390/genes14091783)
Supplement: Supplementary file 1 [file genes-14-01783-s001.zip › Supplementary_Figures_Alcaligenes.pdf]

Supplementary figures

Comparative genomics reveals novel species and insights into biotechnological potential, virulence and resistance of *Alcaligenes*

Francisnei Pedrosa-Silva[a, *], Thiago M. Venancio[a, *]

[a] Laboratório de Química e Função de Proteínas e Peptídeos, Centro de Biociências e Biotecnologia, Universidade Estadual do Norte Fluminense Darcy Ribeiro (UENF), Brazil.

Laboratório de Química e Função de Proteínas e Peptídeos, Centro de Biociências e Biotecnologia, Universidade Estadual do Norte Fluminense Darcy Ribeiro (UENF); Av. Alberto Lamego 2000, P5 / sala 217; Campos dos Goytacazes, Rio de Janeiro, Brazil. TMV: thiago.venancio@gmail.com; FP-S: francisneipedrosa@gmail.com.

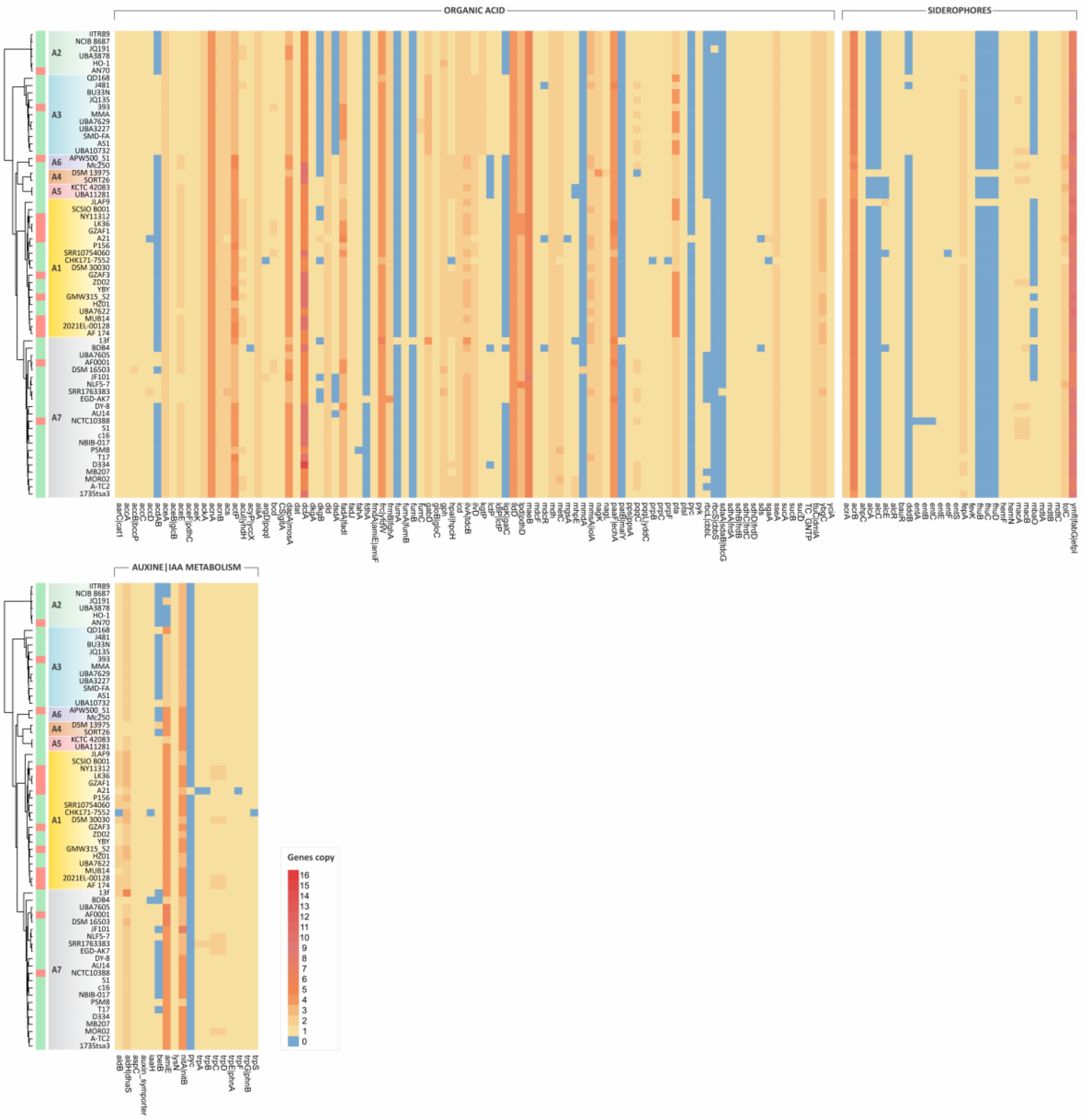

Supplementary figure S1. Distribution of biofertilization genes in *Alcaligenes*. The respective phylogenetic groups of *Alcaligenes* are highlighted in the tree. The heatmaps show the number of genes found for each genome based on PlaBAse annotations.

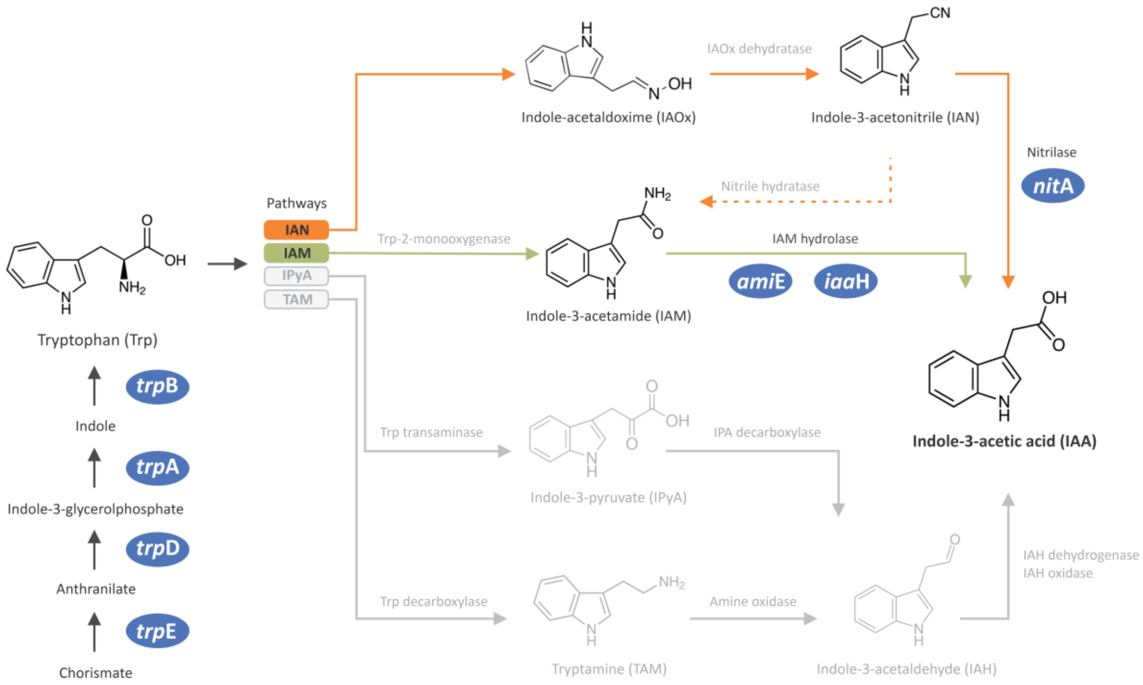

Supplementary figure S2. Deduced pathways for IAA biosynthesis in *Alcaligenes*. The tryptophan (Trp) biosynthetic pathway is shown on the left. Orange and green colors indicate the main IAA biosynthesis pathways. Pathways that were not found are in light-gray (see Supplementary table S1 for details). The names of the pathways indicate the name of their first products. Genes identified are highlighted in blue circles.

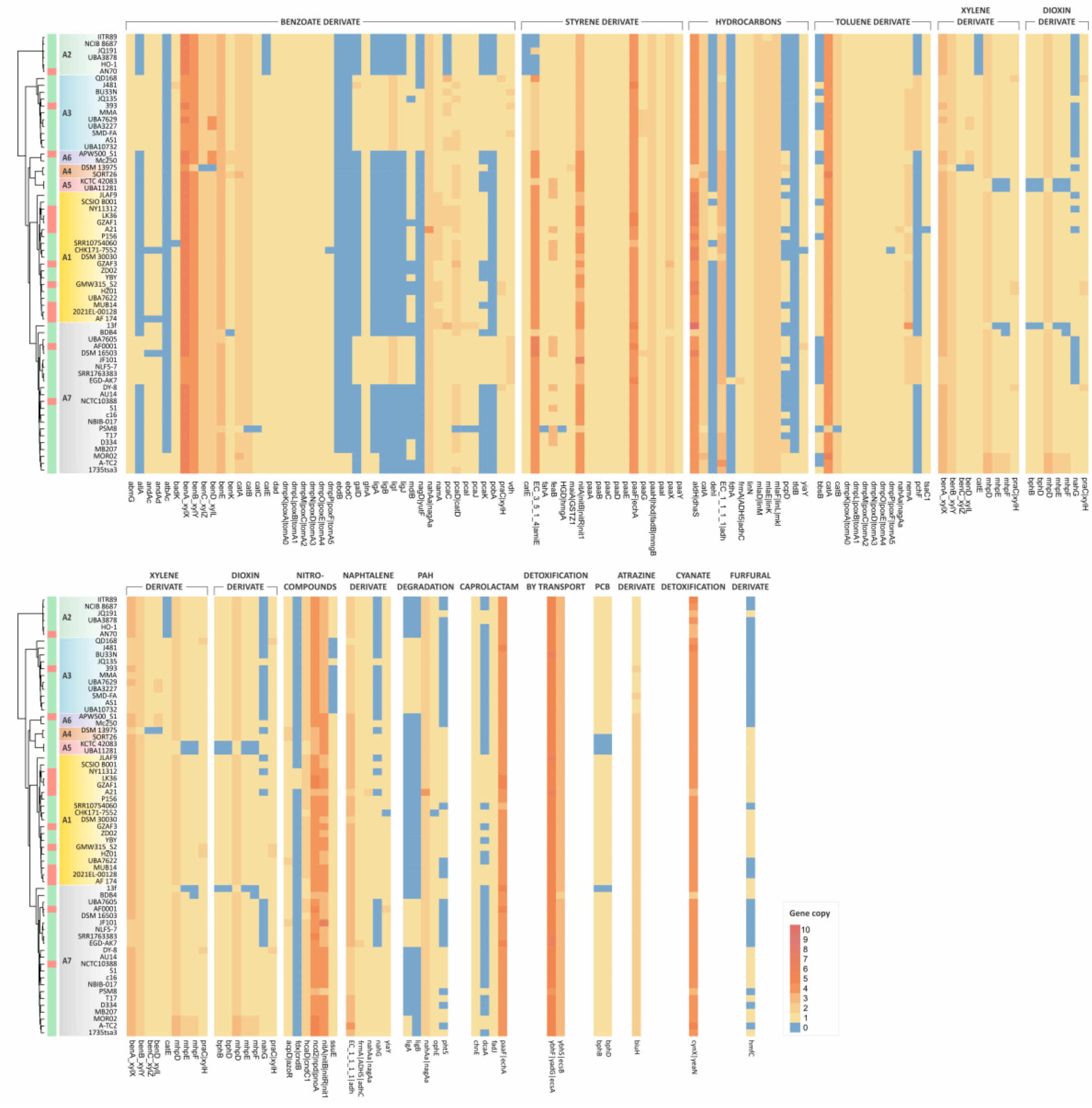

Supplementary figure S3. Distribution of xenobiotic degradation genes in *Alcaligenes*. The respective phylogenetic groups of *Alcaligenes* are highlighted in the tree. The heatmaps show the number of genes found for each genome, based on PlaBAse annotations.

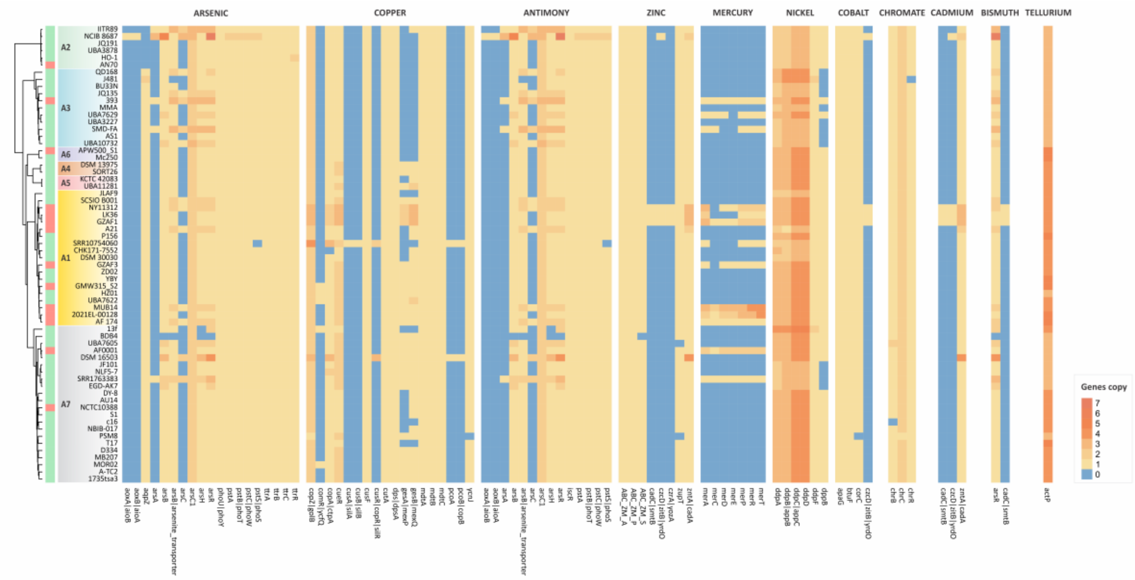

Supplementary figure S4. Distribution of heavy-metal resistance genes in *Alcaligenes*. The respective phylogenetic groups of *Alcaligenes* are highlighted in the tree. The heatmaps show the number of genes found for each genome based on PlaBAse annotations.
